# Allergic diseases in subjects under 18 years living with HIV

**DOI:** 10.1186/1710-1492-10-35

**Published:** 2014-07-07

**Authors:** Leandro S Linhar, Jefferson Traebert, Dayani Galato, Rosemeri M da Silva, Fabiana Schuelter-Trevisol, Natália S Rovaris, Jane da Silva

**Affiliations:** 1 Postgraduate Program in Health Sciences, University of Southern Santa Catarina, Avenida José Acácio Moreira, nº 787, Bairro Dehon, Tubarão, Santa Catarina Postal code: 88704-900, Brazil; 2 Postgraduate Program in Medical Sciences, Federal University of Santa Catarina, Hospital Universitário Polydoro Ernani de São Thiago, Campus Universitário, Trindade, Florianópolis-SC Postal code: 88040-900, Brazil

## Abstract

**Background:**

In recent decades there has been an increase in the prevalence of allergic disease. Manifestations of these diseases have allegedly been observed in people living with Human Immunodeficiency Virus (HIV), however, few studies have been directed at patients under 18 years old. In this context, the aim of this study is to estimate the prevalence of allergic disease in patients under 18 years old, living with HIV, and to investigate the relationship between clinico-immunological characteristics of the HIV infection and atopy.

**Methods:**

This is a cross-sectional epidemiological study involving patients under 18 years of age who were followed up by specialized HIV services in the Southern Region of the State of Santa Catarina, Brazil, from February to October 2012. Data collection tools included a questionnaire established by the International Study of Asthma and Allergy in Childhood (ISAAC), socio-demographic data, as well as laboratory test results obtained from the medical records. Blood samples were taken to measure total serum Immunoglobulin E (IgE) levels and a Radioallergosorbent Test (RAST) for the main aeroallergens. Analysis was performed using Student’s t test, chi-squared, Fisher’s exact and Mann–Whitney tests, wherever indicated, with p < 0.05 value considered significant.

**Results:**

29 individuals were evaluated. The prevalence of symptoms of allergic disease was 65.5% (95% CI 56.1-74.8), the most frequent being rhinitis 44.8% (95% CI 35.0-54.5), followed by asthma 37.9% (95% CI 28.3-47.4) and eczema 27.6% (95% CI 18.8-36.3). RAST was positive in 20.7% of the individuals. There was no significant difference in terms of total serum IgE between individuals with and without symptoms of allergic disease. Nevertheless, a high frequency of raised levels of total serum IgE (40.7%) and an association between raised IgE and clinical staging of disease were observed. A further association between CD8+ cell count and prevalence of symptomatic allergic disease (p = 0.014) was observed.

**Conclusion:**

There was a high prevalence of reported allergic disease, as well as a high frequency of raised levels of total serum IgE. The association between CD8+ cell count and the prevalence of symptomatic allergic disease corroborates studies that demonstrated the role of such cells in the development of allergic disease.

## Background

Clinical manifestations of allergic disease have been reported in individuals living with Human Immunodeficiency Virus (HIV), such as rhinitis, asthma, cutaneous rashes consistent with atopic eczema, symptoms of drug hypersensitivity, and pruritic cutaneous alterations [1-3]. Over the past decades, a global increase has been observed, including in Brazil, in the prevalence of diseases such as asthma, rhinitis and eczema, particularly in pediatric patients [4,5]. The clinical and immunological aspects of HIV-related atopy have been better explored in cross-sectional studies directed at the adult population [6,7]. It seems, however, that atopy in HIV-infected children may be in part modulated by genetic and environmental factors, or even by immunological conditions. It is plausible that reactivity to environmental allergens may undergo positive and negative modifications associated with T-helper 2 (Th2)-linked immune changes [8]. On the other hand, when immunological parameters, such as total serum immunoglobulin E (IgE), are investigated, high levels of this immunoglobulin are found to be related to HIV disease progression [9,10] and the presence of HIV antigen specific IgE [11].

Few international studies are found that specifically address HIV in people under 18 years of age. Considering the diversity of information that can be explored in this population and the characteristics that every study shows, further research is needed on this relevant issue. Thus, the aim of this study was to investigate if symptoms of asthma, rhinitis and eczema are frequent in HIV-infected individuals aged 1 to 18 years, in a community in southern Santa Catarina, Brazil. The purpose was also to evaluate the association between clinical and immunological characteristics of HIV infection and atopy in this population.

## Methods

### Study type, location and sample

A cross-sectional epidemiological study was performed on children and adolescents, aged 1 to 18 years, from 18 municipalities that form the Association of Municipalities of Laguna Region (AMUREL), located in southern Santa Catarina, Brazil [12].

The sample population consisted of 36 individuals living with HIV, aged between 1 and 18 years, under regular follow-up at different specialized HIV services in the region.

Individuals with a confirmed diagnosis of HIV, with at least four months of outpatient follow-up, were included. Individuals were excluded when their parents or guardians were not interested in taking part in the study or declined to sign an informed consent form, or if the participants were using therapeutic drugs that could interfere with the laboratory results, such immunomodulators, corticosteroids, chloroquine and hyperimmune gamma globulin.

### Data collection tools

The International Study of Asthma and Allergies in Childhood (ISAAC) questionnaire was used to investigate allergic disease symptoms (asthma, rhinitis and eczema), which was duly translated into Portuguese and validated in Brazil [13-15]. The diagnosis of asthma was based on the occurrence of one of the following: four or more wheezing attacks in the past year; one to three wheezing attacks combined with sleep interruption due to wheezing in the past year; or one to three attacks, without sleep disturbance, but wheezing following physical exercise and nocturnal dry cough [16].

The diagnosis of allergic rhinitis was based on symptoms of sneezing and rhinorrhea in the past 12 months, or the presence of itchy-watery eyes with a history of rhinitis at some time in their life [14].

Eczema was defined as itchy skin patch in flexural areas, which has waxed and waned over the past 12 months, and having eczema at some point during their lifetime [15].

Additionally, socioeconomic and demographic data, as well as laboratory test results were collected from the medical records to complete the investigation.

### Supplementary laboratory data

On the day of viral load and CD4+ T-lymphocyte subpopulation count sample collection, an additional blood sample was taken for the titration of total serum IgE and radioallergosorbent test (RAST). Total serum IgE measurement was performed using the chemiluminescence immunoassay method, for which the detection limits varied according to the age of the individual, i.e., 1 to 4 years, 351.6 UI/mL; 5 to 10 years, 393.0 UI/mL; 11 to 15 years, 170.0 UI/mL; and over 15 years of age, 165.3 UI/mL.

RAST was performed using the fluoroimmunoassay technique (ImmunoCap) for the following allergens: dust mites, cockroaches, animal epithelia, feathers, and fungi. The degree of sensitization ranged from low to very high (classes 0 to 6), according to the concentration (KU/L) of specific IgE to any of the allergens tested.

### Statistical analysis

The collected data were stored on a database and analyzed using GraphPad Prism 6. The D’Agostino test was applied to evaluate the normal distribution for the studied variables. Quantitative variables were recorded as mean and standard deviation in the case of a normal distribution, and as the median with minimum and maximum values when the distribution was skewed. Qualitative variables were presented as absolute values and proportions. Student’s t test, chi-squared or Fisher’s exact and Mann–Whitney tests were used according to the nature of the variables studied, and were considered significant when p < 0.05.

The Research Ethics Committee of the University of Southern Santa Catarina (UNISUL) approved this study (code number 11.060.4.01.III).

## Results

**Figure 1:**
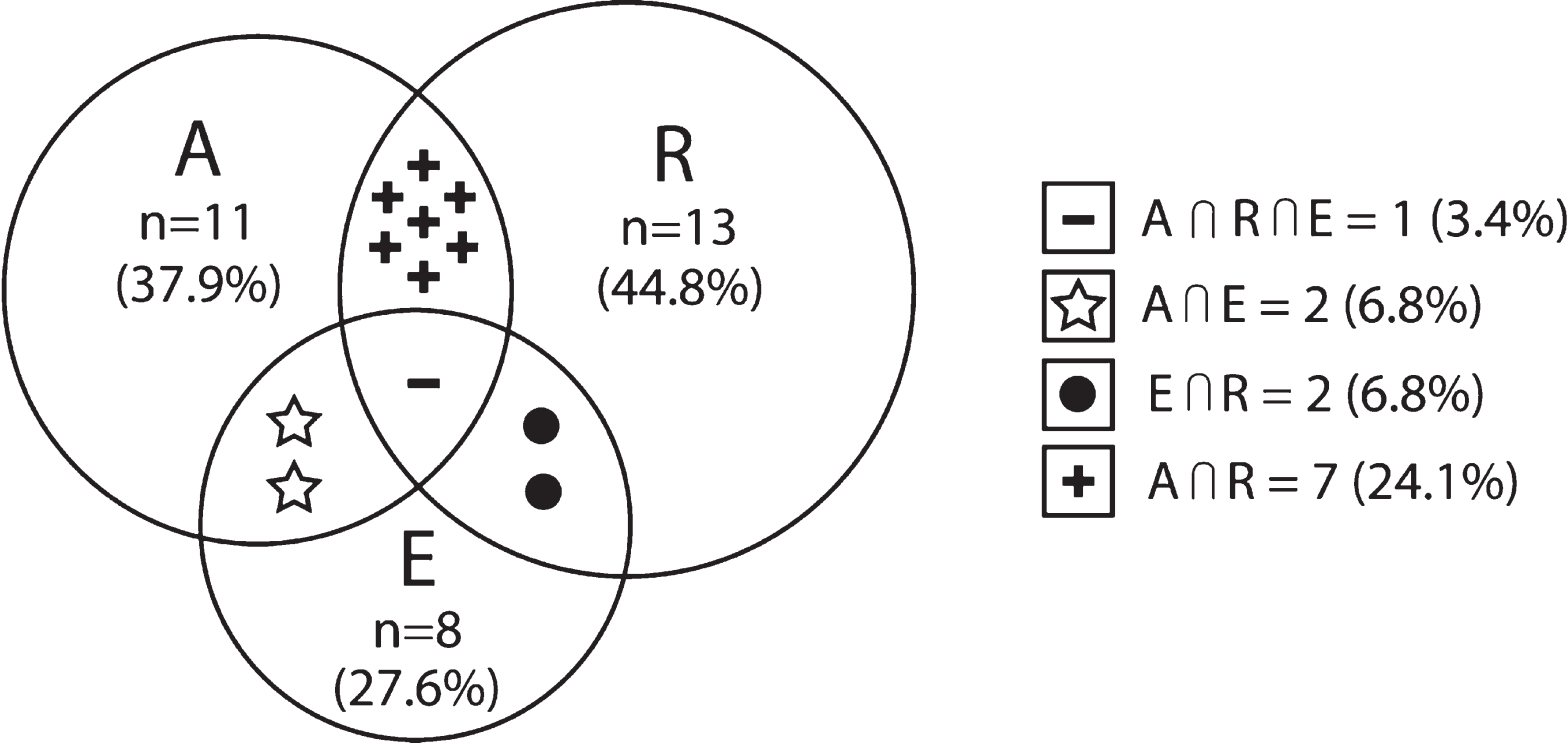
**Distribution of young people (aged 1–18 years) living with HIV and frequency of reported asthma (A), rhinitis (R) and eczema (E), according to the allergic disease questionnaire.** ∩ = Overlap.

Twenty-nine individuals were evaluated. The response rate was 80.5%. Seven patients did not show interest in participating in the study, and were excluded. No patients were using immunomodulators, corticosteroids, chloroquine or hyperimmune gamma globulin. The quantitative and qualitative data of the study population are presented in Tables 1 and 2, respectively.Based on the responses to the ISAAC questionnaire, 19 individuals confirmed that they had allergic diseases, which generated a prevalence of 65.5% (95% CI 56.1-74.8). Symptoms of rhinitis were reported by 44.8% (95% CI 35.0-54.5). A probable diagnosis of asthma was observed in 37.9% (95% CI 28.3-47.4). Among children under age 3, who can be considered transient wheezers, only one presented with symptoms of asthma. Symptoms of eczema were reported by 27.6% (95%CI 18.8-36.3). Figure 1 illustrates the distribution of the studied allergic diseases.

**Table 1:** Characteristics of the individuals living with HIV: quantitative data

**Order**	**Age (years)**	**Time with HIV (months)**	**CD4+ cells/mm**^**3**^	**CD8+ cells/mm**^**3**^	**CD4+/ CD8+**	**Viral load copies/ml**	**Eosinophils**		**Total IgE Ul/ml**
**n**							**%**	**Abs. mm**^**3**^	
1	16	66	552	743	0.74	<50	1	34	25
2	17	5	633	1743	0.36	<50	2	156	282
3	13	105	293	1132	0.25	20434	1	35	135
4	3	8	1220	1394	0.87	8141	NA	NA	547
5	13	150	628	1511	0.41	2489	2	160	493
6	7	86	916	774	1.18	151	NA	NA	57
7	12	46	707	1528	0.46	7486	14	868	813
8	3	26	1323	1073	1.23	<50	2	148	31
9	14	11	234	1079	0.21	<50	NA	NA	54
10	12	97	525	858	0.61	1437	5	279	NA
11	8	74	845	1190	0.71	<50	NA	NA	NA
12	4	48	672	1277	0.52	35411	NA	NA	49
13	11	107	236	2888	0.08	116038	3	207	30
14	8	95	820	4064	0.20	50179	2	154	28
15	16	22	524	1963	0.26	15001	6	372	70
16	1	6	2474	2193	1.12	54165	NA	NA	319
17	12	72	1146	1026	1.11	<50	NA	NA	592
18	13	156	983	980	1.00	<50	NA	NA	186
19	4	20	418	692	0.60	<50	NA	NA	304
20	14	174	1099	1137	0.96	50	2	122	25
21	13	117	346	2225	0.15	50	NA	NA	242
22	12	117	571	577	0.98	335	NA	NA	42
23	7	60	1913	1266	1.51	50	3	204	128
24	6	16	468	638	0.73	2008	NA	NA	382
25	10	117	1173	948	1.23	50	1	82	13
26	13	160	807	896	0.90	125	2	110	380
27	8	19	593	1607	0.36	2671	NA	NA	802
28	12	157	974	668	1.45	400	NA	NA	1217
29	7	18	974	3439	0.28	50	NA	NA	609
Median	12	72	707	1137	0.71	2008	2	155	186
Mean	9.96	74.4	829.8	1431.3	0.71	15081.9	3.3	209.3	290.9
SD	4.32	54.2	488.1	842.4	0.41	28441.5	3.4	209.7	308.5

AMUREL - SC, 2012.

Abs: Absolute Number; Max: maximum; Min: minimum; NA: Not Available, SD: standard deviation.

**Table 2:** Characteristics of the individuals living with HIV: qualitative data

**Order**	**Ethnicity**	**Sex**	**Clinical stage**	**Immunological stage**	**RAST**	**Antiretroviral therapy**
**n**						
1	NCa	M	N	1	-	Y
2	Ca	M	N	1	-	N
3	Ca	M	N	2	-	Y
4	Ca	F	B	1	-	Y
5	NCa	F	A	1	+	Y
6	NCa	F	N	1	+	Y
7	Ca	F	B	1	-	Y
8	Ca	M	C	1	-	Y
9	Ca	M	B	2	+	Y
10	Ca	M	B	1	-	Y
11	Ca	F	B	1	-	Y
12	Ca	F	C	2	-	Y
13	Ca	M	B	2	-	Y
14	NCa	M	N	1	-	N
15	NCa	M	B	1	-	N
16	NCa	F	N	1	-	Y
17	Ca	F	N	1	-	Y
18	Ca	F	B	1	-	Y
19	NCa	F	N	1	-	Y
20	Ca	M	N	1	-	Y
21	Ca	M	B	2	-	Y
22	Ca	F	A	1	-	Y
23	Ca	F	N	1	+	Y
24	Ca	M	A	2	-	N
25	Ca	F	N	1	-	Y
26	Ca	F	A	1	+	Y
27	Ca	F	A	1	-	N
28	NCa	F	A	1	+	Y
29	Ca	F	N	1	-	Y

AMUREL - SC, 2012.

Ca: Caucasian, NCa: Non-caucasian; M: Male, F: Female; N: Asymptomatic A: Mild signs and/or symptoms, B: Moderate signs and/or symptoms, C: Severe signs and/or symptoms; 1: Absent, 2: Moderate, 3: Severe; Y: Yes, N: No; RAST: Radioallergoabsorbent Test.

The socio-demographic characteristics of the individuals living with HIV were evaluated. There was no significant difference between the groups (“yes” *vs.* “no” responses) regarding age, gender, ethnicity, family income and number of people per household. Similarly, there was no difference between the groups regarding the use of HAART.

Table 3 shows the characteristics of the individuals living with HIV, regarding their blood test results and reported allergic diseases.

**Table 3:** Blood sample characteristics of the individuals living with HIV, according to their reported allergic diseases

**Blood sample results**	**Answered “yes” to allergic disease**	**Answered “no” to allergic disease**	***p-*** **value**
	**n = 19 (65.5%)**	**n = 10 (34.5%)**	
	**Median (Min-Max) or Mean ± SD**	**Median (Min-Max) or Mean ± SD**	
Eosinophils (Abs.)	180 (110–868)	115 (34–279)	0.141
Eosinophils (%)	2.5 (2–14)	1.5 (1–5)	0.068
Viral load (copies/ml)	50 (50–3541)	367.5 (50–8141)	0.613
IgE T (UI/mL)	262 (25–813)	57 (13–1217)	0.410
CD4+ (cells/mm^3^)	707 (234–2474)	772 (293–1323)	0.679
CD8 + (cells/mm^3^)	1277 (638–4064)	903 (577–1511)	**0.014**
CD4+/CD8+ ratio	0.61 ± 0.09	0.90 ± 0.15	0.073

(n = 29). Boldface reflects statistical significant difference.

## Discussion

Given that 7 patients refused to participate in the study, it was possible to evaluate 29 individuals. Patients living with HIV and some health professionals were concerned about the security of personal information provided to the researchers, despite clarification of the research purpose and assurance of anonymity. That was the most common reason underlying the refusal to participate in the study.

In this population, a high prevalence of reported allergic disease was observed, with 65.5% (95% CI 56.1-74.8) of the individuals reporting symptoms for some of the allergic diseases investigated. The most frequent symptom was rhinitis, followed by asthma and eczema. Studies have shown a prevalence of allergic disease in the pediatric population with HIV that ranged from 10% to 52% [8,9,17-19]. These studies used various methods to diagnose allergy, including atopy investigation using total IgE titration [8,17,18], RAST [18] and/or skin patch testing for allergens [8,9,19].

The high prevalence found in the present study may be due to the method used to investigate allergy. The ISAAC questionnaire was selected for its standardization, validation in Brazil, and its high sensitivity and specificity in detecting asthma, rhinitis and eczema [13-15], in addition to its ease of use. The fact that this method aims to identify disease based on reporting of symptoms rather than on medical history and clinical examination, combined with allergy tests and/or serum IgE testing, may have overestimated the prevalence of allergic disease in this population. The application of the ISAAC questionnaire in Brazil revealed that the prevalence rates of allergic diseases among schoolchildren aged 6-7years and 13–14 years were lower than those found in our study [4].

A high prevalence of symptoms of asthma, rhinitis and eczema in individuals living with HIV has been reported [20]. In a study that evaluated self-reported symptoms of rhinitis in HIV outpatients, a high prevalence (80% of the study population) of the disease was reported [21]. Amongst children and adolescents living with HIV, the history of rhinitis was estimated at 60% [19]. Asthma was also considered a disease of high incidence and prevalence among children and adolescents living with HIV, according to other studies [22-24]. Regarding skin manifestations of HIV infection, eczema has been reported as a rather frequent disease among adults and children [25-29]. In a study performed on hospital HIV inpatients and outpatients, a clinical evaluation of 796 cases revealed that 19.2% of them had eczema [27]. It was found to be the most frequent type of dermatitis among 70 children with HIV treated at the Tropical Medicine Foundation of Amazonas, detected in 22.9% [26]. These studies included a clinical evaluation of the patients; therefore, they may provide more reliable information on the true prevalence of eczema in children with HIV. Nonetheless, the frequency of 27.6% obtained in the present study via reporting of symptoms was close to that number.

Skin tests, as well as allergen-specific IgE via RAST, have similar sensitivity and diagnostic value in the investigation of allergic diseases [30,31]. The advantages of the skin test are its immediate result and low cost. It does, however, require an appropriately trained professional to perform and interpret the results, which was not available at the health units involved in this study. Therefore, RAST was the preferred method. RAST-positive individuals accounted for 20.7% of the cases, and 6.9% reported symptoms of allergic disease. However, no significant difference was found when compared with asymptomatic individuals (13.8%). The presence of serum IgE or a positive skin test denotes a status of sensitization. The detection of sensitivity to an allergen, however, is not equivalent to a clinical diagnosis, hence the importance of a medical history and knowledge of the specific characteristics of the disease when choosing and interpreting test results [32].

There was no significant difference between individuals in terms of total IgE levels. Furthermore, when patients were divided into groups of raised and normal IgE levels, the prevalence of allergic disease was not significantly different. An increase in IgE levels does not, in fact, translate into an increase in the prevalence of allergic disease in the context of HIV infection [33]. Although raised total serum IgE may provide information that corroborates the diagnosis of atopic disease, in HIV cases this interpretation is more complex due to cell stimulated IgE production by B lymphocytes, i.e., T CD4+ lymphocytes or Th are compromised in both quantity and function. Evaluation of changes in cytokine profiles for Th1 and Th2 following interaction with viral proteins is advised to account for the raised levels of total IgE found in HIV patients [34]. Some studies have identified high levels of total IgE by age in some HIV-infected children [11,17,18], including the presence of specific IgE against HIV antigens [11]. These data suggest that raised total IgE levels in individuals living with HIV, aged up to 18 years, as well as in some adults living with HIV [33], may be due to non-specific polyclonal activation, which is typical of such infection, or in some cases, as a result of the production of HIV-specific IgE [11,33].

There was a high frequency of raised total IgE in 40.7% of our study population, which corroborates the findings of other studies that used pediatric populations with HIV [8,11,17,18]. In addition, the groups with raised IgE levels presented more clinically advanced stages than the normal IgE group; however, no difference was detected in terms of immunological staging. Data from the literature suggest that there is a direct link between raised IgE levels and disease progression, both in adults [33] and children [9,10]. An abnormal synthesis of IgE during HIV infection may be explained by the change in the cytokine profile for Th1 and Th2 as the disease progresses. Cytokines such as interferon gamma (IFN-y), from the Th1 profile, and interleukin 4 (IL-4), from the Th2 profile, undergo changes during HIV infection, in which a lower concentration of the latter and higher concentration of the former is observed. The Th2 profile favors the IgE synthesis, whereas viral proteins appear to stimulate IgE production by inhibiting the Th1 profile, which inhibits Th2. These changes may become pronounced in the course of HIV infection [34].

As per other studies performed on individuals aged under 18 years, this study observed that raised IgE levels were only related to the clinical stage of disease, which ranged from absent to severe signs and symptoms. The defective CD4+ cell function may occur independently from the clinical stage of HIV disease [35]. Therefore, the overproduction of IgE in such individuals may be a reflection of altered CD4+ cell function. Serial IgE measurements in such individuals may be a simple and low-cost method for monitoring disease progression and confirming the association detected by such results.

An interesting finding was the significant association between the CD8+ count and the prevalence of symptoms of allergic disease (p = 0.014). Published studies have highlighted the role of CD8+ cells on the physiopathology of allergic diseases, such as asthma [36,37], eczema [38], and more recently, rhinitis [39,40]. They report similar roles for CD8+ and CD4+ cells via the production of cytokines, such as IL-4, or other inflammatory mediators, which is typical of allergic disease. On the other hand, lower level of CD8+ cells and higher rates of asthma can be seen in HIV children undergoing HAART than in those who do not use HAART [22]. It is possible that the use of HAART influences the development of asthma, but how the low level of CD8+ cells can have an influence in this case is still unknown. In the current study, 6 out of 11 patients with probable diagnosis of asthma were using HAART, and the amount of CD8+ cells ranged from 236 cells/mm^3^ to 707 cells/mm^3^, whereas in those who did not use HAART it ranged from 418 cells/mm^3^ to 2474 cells/mm^3^ (data not shown).

The literature review did not reveal any published study that has associated CD8+ count and prevalence of allergic disease in patients living with HIV. It does, however, seem logical that there should be an association between CD8+ cells and raised IgE levels in HIV infection [41-43]. CD8+ cells could induce IgE synthesis, even in individuals with a low CD4+ count, due to a subtype of CD8+ cells, similar to Th2 cells, known as T-cytotoxic 2 (Tc2), and produce cytokines that stimulate the synthesis of IgE, such as IL-4 [41]. The action of these CD8+ cells may also occur in allergic diseases [44], although in our study population no association was detected between IgE levels and CD8+ count. It is possible that these cells have played a role at the onset of the allergic disease, via the release of other mediators that lead to allergic inflammation.

A limitation of the study was the relatively small sample size. A robust statistical analysis was not possible to perform due to the small number of patients, as well as a few losses that occurred during the follow-up period. It should be noted that this appears to be a common issue, which has been demonstrated by several other studies on children and adolescents living with HIV [9,11,17-19]. Another limitation was the lack of a control group of individuals without HIV disease, which would help clarify the relationship between HIV infection and allergic diseases. The study population was limited to HIV treatment centers, where no uninfected subject was found, thus rendering this study into descriptive traits. For that purpose, further studies should be performed on individuals aged less than 18 years living with HIV, preferably in a longitudinal follow-up study, with a control group and large sample.

## Conclusion

Symptoms of allergic disease, especially rhinitis, were often reported by individuals aged 18 years and under living with HIV. Furthermore, there was no difference between allergic and non-allergic patients regarding the presence of atopy, as evaluated by RAST and total serum IgE levels; however, a high prevalence of raised IgE levels was observed in some individuals who presented a significantly more advanced clinical stage of the disease than those with normal IgE. Finally, the association between CD8+ count and the prevalence of symptoms of allergic disease (despite the limitations of sample size) corroborates what has been reported in the literature, which suggests that CD8+ cells play a role in developing these diseases, including in the context of HIV infection.

## Competing interests

The authors have no conflicts of interest to disclose.

## Authors’ contributions

JS, JT, DG, RMS, FST contributed to the conception and design of the study, revision of data analysis and critical revision of the manuscript. LSL and NSR performed the data collection and conducted the analysis and interpretation of the data. LSL and JS wrote the manuscript. JS had the primary responsibility for the final content. All authors have read and approved the manuscript as submitted.
